# Pospiviroid Infection of Tomato Regulates the Expression of Genes Involved in Flower and Fruit Development

**DOI:** 10.3390/v10100516

**Published:** 2018-09-21

**Authors:** Katia Aviña-Padilla, Rafael Rivera-Bustamante, Natalia Y. Kovalskaya, Rosemarie W. Hammond

**Affiliations:** 1 Campus Juriquilla, Universidad Nacional Autónoma de Mexico, Querátaro Qro 76300, Mexico; ib.katia@gmail.com; 2 Centro de Investigación y de Estudios Avanzados del IPN, Unidad Irapuato, Irapuato, Guanajuato 36821, Mexico; rrivera@ira.cinvestav.mx; 3 United States Department of Agriculture, Agricultural Research Service, Molecular Plant Pathology Laboratory, Beltsville, MD 20705, USA; natalia.kovalskaya@ars.usda.gov

**Keywords:** viroid pathogenicity, tomato planta macho viroid, Mexican papita viroid, tomato fruit development, hormone regulation, RNA-mediated gene regulation, flower development

## Abstract

Viroids are unencapsidated, single-stranded, covalently-closed circular, highly structured, noncoding RNAs of 239–401 nucleotides that cause disease in several economically important crop plants. In tomato (*Solanum lycopersicum* cv. Rutgers), symptoms of pospiviroid infection include stunting, reduced vigor, flower abortion, and reduced size and number of fruits, resulting in significant crop losses. Dramatic alterations in plant development triggered by viroid infection are the result of differential gene expression; in our study, we focused on the effect of tomato planta macho viroid (TPMVd) and Mexican papita viroid (MPVd) infection on gene networks associated with the regulation of flower and fruit development. The expression of several of the genes were previously reported to be affected by viroid infection, but two genes not previously studied were included. Changes in gene expression of *SlBIGPETAL1* (bHLH transcription factor) and *SlOVA6* (proline-like tRNA synthetase) are involved in petal morphology and fertility, respectively. Expression of *SlOVA6* was down-regulated in flowers of TPMVd- and MPVd-infected plants, while expression of *SlBIGPETAL1* was up-regulated in flowers. Up-regulation of *SlBIGPETAL1* and down-regulation of *SlOVA6* were positively correlated with symptoms such as reduced petal size and flower abortion. Expression analysis of additional tomato genes and a prediction of a global network association of genes involved in flower and fruit development and impacted by viroid infection may further elucidate the pathways underlying viroid pathogenicity.

## 1. Introduction

Viroids are small, infectious RNAs that cause economically important diseases in many crop plants [1,2]. As the smallest known agents of infectious disease (246–401 nucleotides, nt), they are composed of a highly-structured, single-stranded circular naked and non-coding RNA genome. Despite their lack of mRNA-coding capacity, they replicate autonomously in their plant hosts by utilizing plant-encoded enzymes. In addition to their effect on crop productivity, viroids are of interest as a model system to analyze many aspects of host–pathogen interactions at the metabolic and genomic levels [3,4,5,6,7,8,9].

Viroid systemic infection is commonly associated with the expression of symptoms related to a general delay of plant development (stunting, axillary bud break, leaf epinasty and distortion, stem and veinal necrosis and chlorosis, reduction of flower size, flower abortion, and the reduction and alteration of fruits) [1,6,10,11,12,13,14]. The severity of symptoms depends upon the plant host, the viroid species and viroid strain, and environmental conditions [15,16,17].

Tomato (*Solanum lycopersicum* L.) is an important vegetable crop worldwide and has been used as a model plant for basic research on fruit growth and development. Tomato planta macho viroid (TPMVd; 360 nt) [18,19] and Mexican papita viroid (MPVd; 359 nt) [20], proposed to be members of the same species in the family *Pospiviroidae* [21,22], are severe pathogens on tomato. Li et al. [16] mapped the virulence determinant factor in TPMVd on tomato to a single base pair in the right terminal domain of the viroid RNA (Supplementary Figure S1). Other pospiviroids, including potato spindle tuber viroid (PSTVd), tomato apical stunt viroid (TASVd), and tomato chlorotic dwarf viroid (TCDVd), cause similar symptoms in tomato, with varying degrees of severity [1,2].

The molecular basis of symptom formation in viroid-infected plants is unknown, although the nuclear location of pospiviroids suggests interactions with the host genome and/or transcription factors. Viroid infection does not cause gross changes in nucleic acid metabolism, but increased transcription of defense-related genes has been reported [23,24,25,26,27,28,29]. Using macroarray technology, Itaya et al. [25], Owens et al. [26] and Więsyk et al. [30] observed altered gene expression patterns in viroid-infected plants, including induction or suppression of genes encoding proteins involved in defense and stress responses, cell wall structure, chloroplast function, hormone metabolism and signaling and protein metabolism, among others. Many of the symptoms caused by viroid infection suggest an imbalance in growth hormones. A significant decrease in endogenous gibberellins (GA_3_ and/or GA_1_) was observed in viroid-infected plants [31], and growth reduction in citrus caused by infection with citrus exocortis viroid (CEVd) was correlated with reduced levels of gibberellin 20-oxidase (GA20ox) mRNA [32].

RNA-induced silencing of host genes has been proposed as one mechanism of viroid pathogenesis [9,33,34,35,36,37,38,39,40,41]. As might be expected from their highly base-paired structures and double-stranded RNA replication intermediates, viroid genomes can be processed into small RNAs (sRNAs) by Dicer-like (DCL) enzymes. The viroid sRNAs (vd-sRNAs) guide the RNA-induced silencing complex (RISC) against viroid RNAs and host mRNAs. Structurally similar to endogenous small RNAs in plants, vd-sRNAs range in size from 21–24 nt in length; these small RNAs have been detected, and in some instances characterized, in infected tissues from viroid-host combinations of both viroid families.

In our previous study, a comparative genomics approach was used to predict vd-sRNA gene targets in Arabidopsis [3]. A highly conserved vd-sRNA in the pathogenicity (P) domain of the pospiviroid genome targeted a WD40 repeat family gene (At3g21540) conserved in tomato (*SlWD40-repeat*; SGN_U563134) and that is proposed to play a role in signal transduction, plant growth, and pollen viability. Expression of this gene was down-regulated in TPMVd-infected tomato plants and, using 5′ RLM-RACE, we determined that the *SlWD40-repeat* mRNA is targeted by vd-sRNA for sequence specific degradation [3]. The role of the *SlWD40-repeat* gene in viroid symptomatology remains to be determined.

As our primary research interest is the impact of viroid diseases on agricultural productivity, the goal of the current study was to determine the interplay of a set of genes related to fruit and flower development affected during viroid infection. In a separate study, Adkar-Purushothama et al. [42] recently reported the role of PSTVd-derived sRNAs in early flowering in infected tomato plants and demonstrated that a PSTVd vd-sRNA derived from the left terminal region targets an mRNA encoding FRIGIDA-LIKE protein 3; this gene is one of two that determine flowering behavior in Arabidopsis. Here, we examined changes in the gene expression of a basic helix loop helix (bHLH) transcription factor gene [43,44] *SlBIGPETAL1* (Solyc05006650.2.1; petal morphology) and *SlOVA6* (Solyc01g096870.2.1; fertility) in tomato plants infected with TPMVd and MPVd. A T-DNA insertion in the Arabidopsis *BIGPETAL-1* gene results in *bigpetal1* and results in large petal size [45]. Mutations in the *SlOVA6* homologous Arabidopsis *OVULEABORTION-6* gene results in reduced growth rate, female sterility, and ovule abortion [46,47]. Our results indicated that the expression of both genes is affected by the progression of TPMVd and MPVd infection, a result that agrees with phenotypic symptoms affecting floral size. We then expanded our study to include an examination of the expression of genes and miRNAs involved in the regulation of plant development, and specifically, those that regulate flower and fruit development. A global network of gene associations among those pathways was proposed to inform a putative mechanism of gene expression during viroid infection.

## 2. Materials and Methods

### 2.1. Plant Infection

*S. lycopersicum* cv. Rutgers seeds were germinated in a commercial seed germination mixture. Twelve days’ post-germination, tomato seedlings were mechanically inoculated with viroids at the cotyledon stage (4 biological replicates per viroid strain). Two leaflets were dusted with carborundum and inoculated with dried viroid-infected tomato tissue homogenized in 50 mM KH_2_PO_4_, pH 7.0. The viroids inoculated were TPMVd (GenBank K00817; 19) and the OG1 strain of MPVd (GenBank L78454; 20). The OG1 strain of MPVd shares 86.4% sequence identity to TPMVd, while it shares 99.4% with MPV-M; TPMVd shares 84% sequence identity to MPV-S [16] (Supplementary Figure S1). For comparison, replicates of mock-inoculated plants were processed at the same time. Following inoculation, plants were maintained in greenhouse conditions under high temperature and long day conditions (30 °C, 16 light hours and 8 dark hours) to favor viroid replication and symptom development.

### 2.2. Total RNA and miRNA Isolation and Processing

For viroid detection, leaves of viroid-infected and mock-inoculated tomato plants (at two, four, and six-weeks post inoculation [w.p.i.]) were used for total RNA extraction. Total RNA was extracted from individual freshly collected young leaf tissue, using the RNeasy Mini kit (Qiagen, Valencia, CA, USA) per the manufacturer’s instructions. Tissue samples were dusted with carborundum (silicon carbide grade 100/120) before extraction to facilitate grinding, and the amounts of total RNA preparations were determined by optical density at 260 nm using a Nanodrop 8000 (Nanodrop, Wilmington, DE, USA). For gene expression analysis, total RNA was extracted from the leaf and flower (including flower buds) of both viroid-infected and mock-inoculated control plants using a modified Tri Reagent (Molecular Research Center, Cincinnati, OH, USA)/RNeasy minicolumn (Qiagen) protocol. Two young leaves of each biological replicate per time point were harvested at two, four, and six w.p.i. and immediately processed. RNA samples were digested with RQ1-DNase (Promega, Madison, WI, USA) and were purified using RNeasy mini columns (Qiagen) before reverse-transcription. Isolated RNA samples were eluted in nuclease free water and subsequently stored frozen at −20 °C. miRNAs were isolated from mock-inoculated and TPMVd-inoculated plant tissues at four w.p.i. using the PureLink™ miRNA isolation kit (Invitrogen, Carlsbad, CA, USA) following manufacturer’s instructions. Isolated RNA samples were eluted in nuclease-free water, and RNA concentration was determined analyzed using a Nanodrop 8000 spectrophotometer; RNA integrity was analyzed using the Agilent 2100 Bioanalyzer and Agilent RNA 6000 Nano kit (Agilent Technologies, Cedar Creek, TX, USA) per manufacturer’s instructions. The samples were subsequently stored at −20 °C.

### 2.3. Reverse Transcription-Polymerase Chain Reaction (RT-PCR) and Sequencing and Quantitative RT-PCR (qRT-PCR) for Viroid Titer

For viroid detection, two assays were performed. Reverse transcription-polymerase chain reaction (RT-PCR) reactions to verify the nucleotide sequence of the viroid isolates were performed using the total RNAs extracted above and the Titan One tube RT-PCR System (Roche Molecular Biochemicals, Chicago, IL, USA) per manufacturer’s instructions with the universal pospiviroid primer pair POSPI-FW/POSPI-RE (Supplementary Table S1) [48]. These primers amplified a fragment of the viroid genome annealing to a region highly conserved between these species. RT-PCR products were analyzed by electrophoresis through a 1% TBE agarose gel followed by staining with the SYBR Safe DNA gel stain (Invitrogen) and visualized on an UV-transilluminator. Each RT-PCR analysis was performed on independently prepared RNA extracts from four individual plants. Once amplified, DNA fragments were cloned into the vector pCR™ 4-TOPO^®^ (Invitrogen, Carlsbad, CA, USA) and plasmid DNAs were purified from the bacterial colonies for subsequent nucleotide sequencing to verify the nucleotide sequence of the viroid isolate.

To determine the viroid titer in infected plants, total RNA was amplified from the same samples using the Brilliant^®^ III SYBR^®^ Green quantitative RT-PCR (qRT-PCR) Master Mix (Agilent Technologies, Santa Clara, CA, USA) on the Mx3000P Real-Time System (Stratagene, La Jolla, CA, USA). Each qRT-PCR reaction contained 10 μL of 2× Brilliant^®^ SYBR^®^ Green III qRT-PCR Master Mix, 1 μL of the total RNA, and 2.5 pM of either the POSPI-FW/POSPI-RE primer pair or the EF1α primer pair (Supplementary Table S1) to normalize RNA concentrations. Conditions used for the qRT-PCR reactions were: 50 °C for 10 min, 95 °C for 3 min, followed by 40 cycles of 95 °C, 5 s, 60 °C, for 20 s. The specificity of the amplifications was confirmed by the single peak of dissociation curves of the PCR products.

### 2.4. Gene Expression Analysis

#### 2.4.1. cDNA Synthesis

First-strand cDNA was synthesized using an oligo (dT) primer from 1 μg total RNA and the Advantage RT for PCR cDNA synthesis kit per the manufacturer’s directions (Clontech Laboratories, Inc., Mountain View, CA, USA). The cDNA reaction was diluted to 100 μL with nuclease free sterile water.

#### 2.4.2. Quantitative PCR (qPCR)

Gene expression profiles were analyzed by qPCR with gene-specific primers designed using the Primer3 online software [49,50], or published primers (Supplementary Table S1). The amplification reactions were performed using Brilliant^®^ III SYBR^®^ Green qPCR Master Mix (Agilent Technologies) on the Mx3000P Real-Time System (Stratagene, La Jolla, CA, USA). Each qPCR reaction contained 10 μL of 2× Brilliant^®^ SYBR^®^ Green III qPCR Master Mix, 1 μL of the diluted cDNA, and 2.5 pM of each gene-specific primer. Thermal conditions used for the qPCR reactions were: 95 °C for 10 min, followed by 40 cycles of 95 °C, 30 s, 55 °C, for 1 min, and 72 °C for 30 s. Specificity of the amplifications was confirmed by the single peak of dissociation curves of the PCR products.

#### 2.4.3. Stem-Loop Quantitative RT-PCR

miRNAs were reverse-transcribed into first strand cDNA in a 7.5 μL reaction containing 50 nM of a specific reverse stem-loop primer, 1× RT buffer, 0.25 mM of each dNTP, 3 μL of Affinity Script reverse transcriptase (Agilent Technologies), and 0.25 u/μL RNase out (Invitrogen) as described by Chen et al. [51]. The reactions were incubated for 30 min at 16 °C, 30 min at 42 °C, 5 min at 85 °C, then 4 °C. The real-time PCR reactions were performed using the Brilliant III Ultra-Fast SYBR QPCR kit (Agilent Technologies). The 10 μL PCR reaction included 1 μL of RT product, 1.5 μM forward primer, 0.7 μM universal primer and 5 μL of 2× SYBR Green PCR mix. The primer sequences are listed in Supplementary Table 1. The reactions were incubated at 95 °C, 10 min, followed by 40 cycles of 95 °C 15 s and 60 °C 1 min. Specificity of the amplifications was confirmed by the single peak of dissociation curves of the PCR products. All reactions were run in triplicate.

#### 2.4.4. Analysis of qPCR Data

Relative quantitation of the target gene transcripts was carried out using the ΔΔ*C*_t_ method. Gene expression fold changes were relative to healthy controls and relative transcript expression levels of each target were normalized with respect to the with *EF-1α* gene (Accession No. X14449) for genes and *U6snRNA* for miRNAs as the internal reference (Supplementary Table S1). The *EF-1α* gene was found to more stable as a reference gene for qRT-PCR analyses and exhibited greater efficiency (data not shown and [52]. Experiments were technically repeated three times, and three independent biological samples were used for each repeat experiment (*n* = 9). Data were analyzed using the MxPro software (Stratagene, San Diego, CA, USA). Specificity of the amplifications was confirmed by the single-peak melting curves of the PCR products. Data were processed in Microsoft Excel and evaluated with R package software [53].

## 3. Results and Discussion

**Figure 1:**
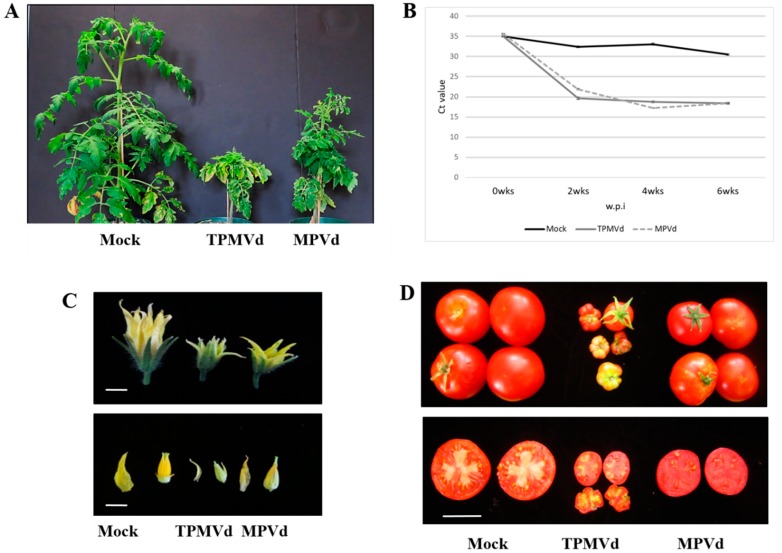
Phenotypes of mock-inoculated and viroid-infected Rutgers tomato plants. (**A**) Tomato plants displaying symptoms of TPMVd and MPVd infection six weeks’ post-inoculation compared to mock-inoculated plants. (**B**) Time course comparison of TPMVd and MPVd-infected vs. mock-inoculated tomato plants. Quantitative reverse transcription-polymerase chain reaction (qRT-PCR) detection (Ct values) using pospiviroid primers during three infection stages as described in Material and Methods. Early infection, 1-2 wpi, middle infection at 4 wpi, and late infection at 6 wpi. (**C**) Comparative size of flowers from mock-inoculated, TPMVd-, and MPVd-inoculated tomato plants (upper) and sepals and carpels dissected from the flowers (lower). Bars = 1 cm. (**D**) Mature fruits of mock-inoculated plants compared to plants inoculated with TPMVd and MPVd (upper) and cross-sections of mature fruits showing reduced locule number and numbers of seeds in viroid-infected versus mock-inoculated fruit (lower). Bar = 6 cm.

Tomato plants (*S. lycopersicum* var. Rutgers) established under greenhouse conditions, exhibited symptoms of stunting, epinasty and chlorosis of young leaves when infected with TPMVd and MPVd (Figure 1A). The OGI strain of MPVd-infected tomato displayed similar symptoms to, but milder than, those of TPMVd (Figure 1A). Although the symptoms of MPVd infection were milder than those of TPMVd, the rates of systemic viroid accumulation were similar from 2 to 6 w.p.i (Figure 1B), suggesting that sequence differences and not viroid titer are responsible for the differences in symptomatology. In reproductive organs, viroid infection resulted in smaller flowers and fruits (mock-inoculated, 41 ± 8.9 g; TPMVd, 9.6 ± 6.7 g; MPVd, 21 ± 6.97 g), with reduced locule size and locule number, and diminished seed set (not shown) (Figure 1C,D).

### 3.1. Gene Expression Changes Associated with Floral Phenotypes

Time course analyses of changes in gene expression of *SlBIGPETAL1* and *SlOVA6* were examined: (a) Early Infection (EI; 1-2 wpi) when symptoms were not visible and viroid replication was not detected; (b) Middle Infection (MI; 3-4 wpi) when plants started to show symptoms and viroid titers were easily detected; and (c) Late Infection (LI; 6 wpi) when plants showed strong symptoms of viroid infection (Figure 1B).

**Figure 2:**
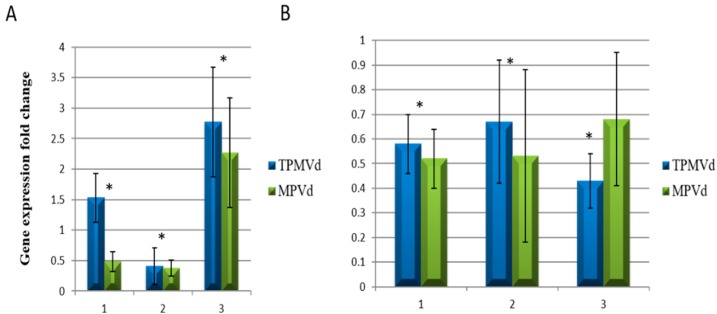
*SlBIGPETAL1* and *SlOVA6* time course gene expression comparison of TPMVd and MPVd-infected *vs* mock-inoculated tomato plants. (**A**) *SlBIGPETAL1* and (**B**) *SlOVA6* gene expression during three infection stages: 1, early infection; 2, middle infection and 3, late infection. Gene expression values are relative to mock-inoculated controls to which a value of 1 was assigned. The *EF-1α* elongation factor gene was used as an internal reference. Error bars indicate two times the value of the SD for the corresponding data set. Asterisks indicate statistical significance when analyzed using a paired *t*-test (*p* < 0.05). Where both treatments are statistically significant from mock-inoculated controls, one asterisk is placed above the bars.

qRT-PCR analysis of *SlBIGPETAL1* and *SlOVA6* gene expression in whole plantlets revealed increased expression for both genes when plants were infected with TPMVd as compared to mock-inoculated controls; these changes in expression were also correlated with plant symptoms and viroid titer during MPVd infection (Figure 1B). In the case of *SlBIGPETAL1*, expression was generally higher in TPMVd compared to MPVd infection, and higher at EI as compared to MI, but later dramatically increased at LI as compared to MI (Figure 2A). By contrast, expression of *SlOVA6* was equivalent under both infective conditions, with a slight decrease in expression at MI as compared to EI under TPMVd infection (Figure 2B). These results indicate that the expression of both genes is affected by the progression of viroid infection.

Previous reports in Arabidopsis have shown that the two *BIGPETAL1 (BPE)* genes are differentially expressed by alternative transcript splicing. *BPEub* [TAIR: At1g59640.1] is ubiquitously expressed and is not directly related to flower development, while *BPEp* [TAIR: At1g59640.2] is mainly expressed in flowers, particularly in petals, and its role in petal size and morphogenesis has been confirmed [45]. To distinguish which of the *Arabidopsis thaliana* variants could be more related to the tomato homologous gene, an expression analysis comparing leaf tissue and flower tissue was performed.

**Figure 3:**
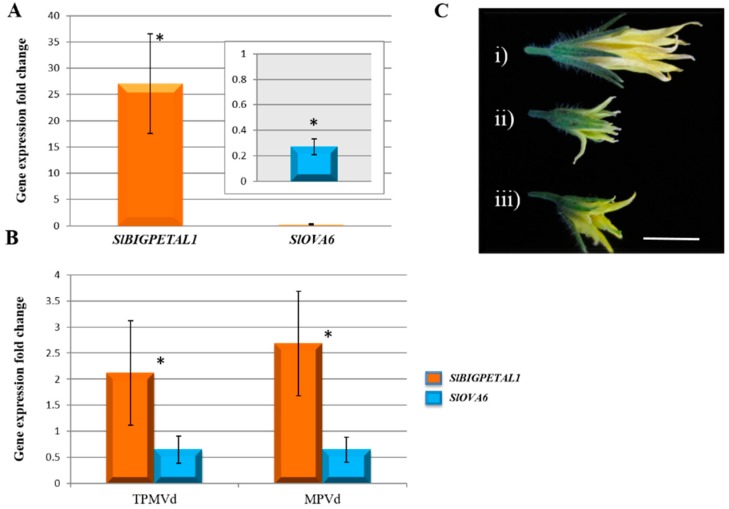
*SlBIGPETAL1* and *SlOVA6* flowering-related genes. *SlBIGPETAL1* and *SlOVA6* gene expression in flowers (**A**) compared to leaves (**B**) in TPMVd and MPVd viroid-infected flower tissue. (**C**) Comparative size of flowers (i) mock-inoculated, (ii) TPMVd and (iii) MPVd-infected flowers. Bar = 1 cm. Gene expression values are relative to mock-inoculated controls to which a value of 1 was assigned. The *EF-1α* elongation factor gene was used as an internal reference. Error bars indicate two times the value of the standard deviation (SD) for the corresponding data set. Asterisks indicate statistical significance when analyzed using a paired *t*-test (*p* < 0.05). Where both treatments are statistically significant from mock-inoculated controls, one asterisk is placed above the bars.

Our results show that *SlBIGPETAL1* is highly expressed in tomato flower tissue, as predicted for its homologue [At1g59640.2] that is responsible for petal morphogenesis in Arabidopsis, while *SlOVA6* is expressed more highly in leaves than in flowers (Figure 3A,B). Both genes have the same expression profile pattern as their corresponding homologue in Arabidopsis, confirming that they could share similar functions. Mutations in two of these genes in Arabidopsis show obvious phenotypic alterations such as defects in petal morphology or fertility, suggesting both play a role in plant reproduction [45]. Where the expression of *SlBIGPETAL1* was up-regulated in flowers of TPMVd- and MPVd-infected plants, *SlOVA6* was down-regulated in these same organs. Up-regulation of *SlBIGPETAL1* and down-regulation of *SlOVA6* are positively correlated with viroid-derived phenotypic symptoms such as reduced petal size and flower abortion suggesting a functional interaction between viroid and plant RNAs. Our results show that viroid infection in the tomato can lead to specific changes in reproductive gene expression.

Although our results show a link between gene expression, viroid infection, and symptom severity, it is not known if vd-sRNAs play a role in viroid disease progression as the correlation between vd-sRNAs and symptoms is not general for all viroid species [7,54].

Flower development requires an interaction and coordination among several gene networks and transcription factors act as a pivotal component of flower organ morphogenesis. Several viroid species, including both of those used in our studies and their relatives in *Pospiviroidae* family, dramatically block development and size of flowers of infected plants and affect their normal function in plant physiological processes and are present in flower buds and ovules of infected plants [55,56] (Figure 3C). Genes involved in the correct development of floral organs might be directly or indirectly affected by viroid infection by either a direct interaction between vd-sRNAs and their targets in floral tissues, or through an indirect interaction that involves viroid components and regulatory pathways that indirectly affect flower development.

### 3.2. Relative Expression of Additional Tomato Genes That Regulate Plant Growth and Development

A regulatory network for coordinated flower and fruit maturation has been studied in Arabidopsis [57,58] and tomato [59] and includes those genes that are controlled by, or control, the recognition of, or synthesis of, auxin, GAs, and brassinosteroids, such as auxin-response factors (*ARF6* and *ARF8*), *AUX/IAA* transcription factors which regulate the expression of ARFs (e.g., *IAA3, IAA9*), *SlGRAS*, *SlDELLA*, *SlWUS*, *TAG*, *F2.2*, *YABBY*, *APETAL*, and *SlAG6*, among others. In addition, plant development is regulated by endogenous microRNAs (miRNAs) which target several of these genes. We examined the relative expression of these genes in mock-inoculated and viroid-infected tomato leaf and flower tissues and describe the results in more detail below.

#### 3.2.1. Gibberellin Biosynthesis and Signaling

**Figure 4:**
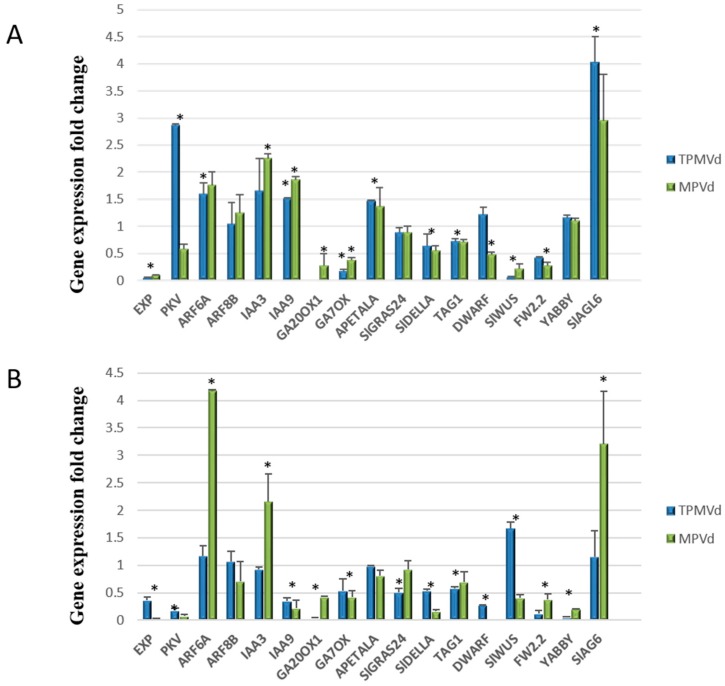
Relative mRNA accumulation of genes involved in leaf, flower, and fruit development in leaves (**A**) and flower buds (**B**) in TPMVd- and MPVd-infected tomato plants. Total leaf RNAs were extracted at 4 w.p.i. Gene expression values are relative to mock-inoculated controls to which a value of 1 was assigned. The *EF-1α* elongation factor gene was used as an internal reference. Data are the means of three biological replications and error bars indicate two times the value of SD for the corresponding data set. Asterisks indicate statistical significance when analyzed using a paired *t*-test (*p* < 0.05). Where both treatments are statistically significant from mock-inoculated controls, one asterisk is placed above the bars.

Several studies indicate that GA biosynthesis and signaling pathways play a pivotal role in viroid pathogenesis. Expression levels of the GA biosynthesis genes, *GA20ox1* and *GA7ox*, previously shown to be down regulated in PSTVd-infected tomato [28] were drastically reduced in leaf and flower tissues in TPMVd- and MPVd-infected plants (Figure 4). Transcription of the *DELLA* gene *GAI* (gibberellic acid insensitive), a key component of GA signaling, was strongly repressed in the stunted in TPMVd- and MPVd-infected plants, confirming the observation of Owens et al. (2012) [28] in PSTVd-infected tomato plants. In our study, the AGCVIIIa protein kinase gene (*pkv*; protein kinase viroid-induced) that was transcriptionally activated in PSTVd-infected tomato and is proposed to be involved in GA signaling [60,61], was also transcriptionally activated in leaves, but not flowers, of viroid-infected plants (Figure 4). In rice, the *YABBY1* gene (encoding a transcription factor) is involved in GA biosynthesis and overexpression resulted in a semi-dwarf phenotype [62]. *YABBY1* also controls carpel number during flower and fruit development [63]. In our analysis, there was no change in expression in leaves of viroid-infected plants, but *YABBY1* expression was severely repressed in flowers (Figure 4B).

#### 3.2.2. Auxin Signaling Pathways

**Figure 5:**
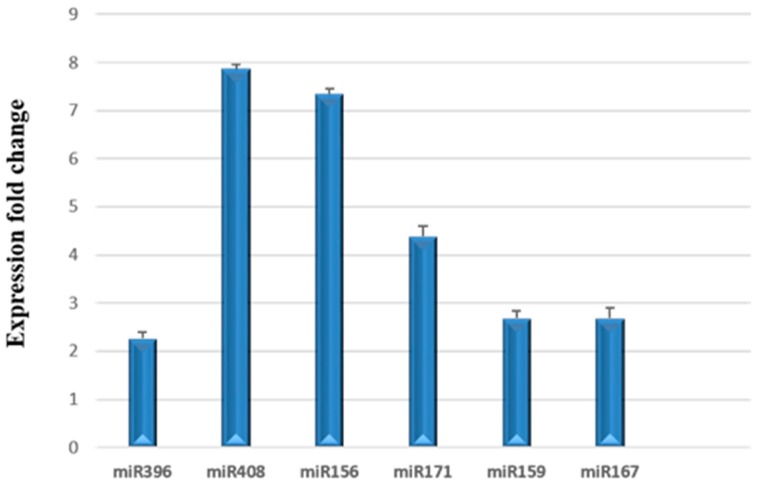
Stem-loop quantitative RT-PCR of miRNA accumulation in TPMVd-infected leaf tissue 4 w. p.i. relative to mock-inoculated controls to which a value of 1 was assigned. *U6snRNA* was used as an internal reference. Error bars indicate two times the value of SD for the corresponding data set. All fold changes were statistically significant from mock-inoculated controls when analyzed using a paired *t*-test (*p* < 0.05).

Auxin-mediated responses/signaling pathways involve *Aux/IAA* genes that encode short-lived nuclear proteins that repress ARFs in response to auxin. ARF proteins bind to cis-regulatory sequenced in the promoters of auxin-dependent genes, controlling their expression and auxin-dependent growth and development. *ARF8* controls petal growth by interacting with the *BIGPETALp* (bpe-p) in Arabidopsis, leading to a reduced response to auxin [64]. When *bpe-p* and *ARF8* are down regulated, the levels of the *Aux/IAA* gene, *IAA9*, increase. In our study, *ARF8a* levels were not significantly different in infected leaves and flower buds when compared to mock-inoculated tissues (Figure 4A,B). *ARF8* is a predicted target of miR167. While we observed a greater than two-fold increase in miR167 in infected tomato leaf tissue (Figure 5), there was not a corresponding decrease in *ARF8a* accumulation. This contrasts with observations made by Zheng et al. [9] whose transcriptome analysis of PSTVd-infected tomato leaf tissue resulted in essentially no expression change in miR167 levels and a two-fold reduction in *ARF8-1* transcripts and that miR167-guided cleavage was enhanced upon viroid infection, leading to suppression of *ARF8* expression.

*ARF6* and *ARF8* regulate growth in reproductive and vegetative tissues, have partially overlapping functions, and are predicted targets of miR167 [65,66]. *ARF6a* was up-regulated in leaves and flower buds of TPMV-infected plants, while *ARF8b* was not changed significantly from when compared to mock-inoculated plants (Figure 4A,B). *SlIAA3*, which intersects auxin and ethylene signal transduction pathways, represses transcription from auxin-responsive (ARF) genes [67]. In our studies, *SlIAA3* was downregulated in TPMVd flowers, with no change in MPVd flowers, and up-regulated in leaves of both TPMVd and MPVd-infected plants. *SlIAA9*, which is involved in fruit development and leaf morphogenesis [68] was up-regulated in leaves and down-regulated in flowers. AUX/IAA-ARF signaling pathways also affect the polarity of auxin gradients and accumulation of auxin in the adaxial side of leaves, resulting in leaf epinasty [69].

#### 3.2.3. Coordinated Signaling among Hormone Pathways

The *DWARF1/DIMINUTO* gene encodes a protein involved in steroid biosynthesis and can affect brassinosteroid levels that affect plant development and fertility [70]. As was shown in potato infected by PSTVd, this gene was down-regulated in viroid-infected leaves and flower buds, and down-regulation in Arabidopsis results in a dwarf phenotype [71]. Expression of this gene is considerably down-regulated in flower buds of tomato infected with MPVd and TPMVd (Figure 4B) and may be associated with the loss of fertility in these organs.

The GRAS family of plant proteins regulates many aspects of plant growth and development, and several members function as transcription factors (TFs) driving downstream transcriptional programs that promote shoot stem cell proliferation e.g., *SlGRAS24* is targeted to the nucleus and is itself a target of miR171 [72]. Overexpression of *SlGRAS24* in tomato resulted in axillary bud emergence, reduced fruit set, arrested fruit and seed development [73], symptoms like tomato infected with TPMVd and MPVd. *SlGRAS24* is also involved in GA and auxin signaling. In our studies, we found that miR171 expression was up-regulated in TPMVd-infected leaf tissue (Figure 4), while *SlGRAS24* expression was little changed in leaves and down-regulated in flower buds (Figure 4A,B, respectively). Because of the phenotypes reported for overexpression of *SlGRAS24*, we assumed that the phenotypes displayed by viroid infection would be the result of up-regulation of genes such as *SlGRAS24*. As this was not the case, additional experiments to dissect the role of miR171 and *SlGRAS24* gene expression in floral and fruit development in viroid-infected tomato will need to be performed.

Carpel number and carpel cell division determine the final size of tomato fruit [58]. *FRUIT WEIGHT 2.2* (*fw2.2*), a gene mapping within the FW2.2 quantitative trait locus that regulates tomato fruit size, functions as a negative regulator of cell division in tomato fruit, potentially by direct interaction with casein kinase II that regulates cell cycle [74]. It has been shown that there is an inverse relationship between fruit size and *fw2.2* gene expression; however, we found that its expression is down-regulated in viroid infected leaf and flower tissues (Figure 4A,B, respectively).

Plant hormones also play a role in organ size control by modulating cell expansion. Expression of the *LeExp2* expansin gene was repressed (Figure 4A,B), confirming results by Qi and Ding [75] who reported that *LeExp2* gene expression was down-regulated in PSTVd-infected tomato plants where stunting resulted from restricted cell expansion, and Martin et al. [13] who reported down-regulation of this gene in CEVd-infected tomato plants.

#### 3.2.4. Transcriptional Control of MADS-Box Transcription Factors and Floral/Fruit Development

Transcriptional control of floral and reproductive development in plants also involves several conserved genes within the MADS-box family of TFs [76]. One effect of the silencing the MADS-box gene *TOMATO AGAMOUS1* (TAG1) in tomato is the increase in non-viable pollen to 55% and reduced seed set, but with no defects in fruit ripening [77]. In viroid-infected tomato, TAG1 expression was reduced in both leaf and flower buds (Figure 4A,B, respectively) suggesting that repression of this gene influences pollen viability and reduced fruit set resulting from viroid infection in tomato.

Of interest is the transcriptional up-regulation of the *SlAG6* gene. *SlAG6 (agamous-like)* is a MADS-box TF that is involved in fruit development following fertilization. When *SlAG6* is present, ovary growth is arrested if not fertilized, resulting in seed abortion [78]. Overexpression of PKV in tobacco results in a significant decrease in viable pollen [61] and silencing of TAG1 also results in an increase in non-viable pollen. The interaction of these pathways may affect fruit development by a combination of arrest of ovary growth, aborted fruit, and male sterility.

*APETALA1* is a MADS-box transcription factor that is involved in leaf and fruit development [79,80]. Floral organs are leaf derivatives and many common factors are involved in the growth and development of these organs [81]. In TPMVd- and MPVd-infected plants, *APETALA1* was slightly up-regulated in leaves and somewhat down-regulated in flowers (Figure 4A,B, respectively). *APETALA1* expression is repressed by the TCP lanceolate transcription factor [79] which was shown to be up-regulated in viroid-infected tomato [33]. Constitutive expression of *APETALA1* in tomato results in a determinate growth habit without affecting fruit yield. Lack of correlation between *APETALA1* expression and symptom phenotypes in our study suggests that this gene may not play a direct role in viroid pathogenesis.

The *WUSCHEL* (*WUS*) gene in Arabidopsis encodes a homeodomain TF that defines the shoot stem cell niche in a complex pathway that requires a cascade of interactions with other transcriptional regulators. Cytokinin signaling activates *WUS* expression during axillary meristem initiation in Arabidopsis and WUS activates the MADS AG TF [82]. In viroid-infected plants, the relative expression of the tomato homolog, *SlWUS*, in leaves and floral buds was dramatically reduced when compared to mock-inoculated plants (Figure 4A,B) and the phenotype of release of shoot apical dominance (axillary shoot development) is like that observed in a recessive *wus* mutant in Arabidopsis [83]. *SlWUS* is also involved in flower and locule development in tomato, and silencing of this gene results in smaller flowers and fruits and alters the expression levels of TAG1 [84], as is observed in viroid-infected tomato.

### 3.3. Relative Expression of miRNAs

Plant miRNAs are small, non-coding RNAs that are produced as discrete sRNA species from MIR genes, are partially complementary to one or more mRNAs, and play crucial roles in integrating genetic networks that regulate development. They function in RNA silencing, translational repression, mRNA cleavage, and post-transcriptional regulation of gene expression. The predicted mRNA targets for several plant miRNAs involved in male sterility and fruit development have been described [85]. In our study, we examined the relative expression of selected miRNAs (miR156, miR159, miR167, miR171, miR396, and miR408) reported to target genes involved in flower, ovule, and anther development present in the miRNA fraction isolated from mock-inoculated and TPMVd-infected tomato leaf tissues (4 w.p.i.). We found that all were up-regulated in response to viroid infection (Figure 5).

Our results contrast with those of Owens et al. [26], who reported that there was a 20-fold decrease in miR156 levels in tomato plants infected with PSTVd, while miR159 levels remained unchanged. Zheng et al. reported a slight repression of miR159 and miR171, no change in miR167, and repression of miR408 in PSTVd intermediate strain-infected tomato leaf tissue 3 w.p.i. when analyzing sequence reads from sRNA sequencing [9]. The contrasting results may be due to specific viroid/host interactions, tissue sampling, time of sampling in viroid infection, or the method used to examine miRNA levels. Owens et al. [26] and Zheng et al. [9] measured miRNA levels using small RNA sequencing and Northern blot analysis, while we used stem-loop qRT-PCR to measure specific miRNAs in miRNA-enriched fractions. Of interest in our study, the levels of miR396, which has been associated with the regulation of expression of bHLH transcription factor expression in tomato fruit development [86], and miR408, which regulates genes involved in leaf development, were up-regulated. As discussed earlier, miR167 has binding sites on *ARF6* and *ARF8* [87] which regulate female and male reproduction in Arabidopsis [88].

## 4. Conclusions

Although the tomato fruit forms late in the plant’s life cycle, its formation and final dimensions are regulated early in the plant’s lifespan. Organ growth and flower development are highly coordinated to give a final, uniform size. Perturbation of these processes results in organ size reduction and flower aberrations. Identifying the target genes, small RNAs and the interacting proteins, that are regulated by RNA-mediated gene expression will help to unravel the network of genes responsible for the characteristic phenotypes displayed in viroid-infected plants [34,89,90].

In Arabidopsis, *BIGPETAL1* interacts with MYB-domain transcription factors and WD-repeat proteins to form complexes involved in cellular differentiation during petal morphogenesis, seed dormancy, ovule and stamen development, and pollen tube growth, with responses being influenced by the action of jasmonic acid signaling [43]. In our previous report, we found that the *SolWD40-repeat* expression was down-regulated in tomato plants infected with TPMVd [3]. Further studies to confirm possible biochemical interactions of the *SlBIGPETAL1* and *SlOVA6* in similar or equivalent protein complexes will be necessary to understand the gene regulatory networks that are responsible for the flowering alterations that prevail in viroid-infected plants.

To gain a better understanding of the results of our gene expression analyses, we generated a predicted network association of genes involved in plant development using STRING (https://string-db.org) and gene ontology (GO) enrichment (Supplementary Figure S2, Supplementary Table S2). The predicted gene regulatory network will serve as a platform for comparative analysis using RNA-seq, transcript profiling, proteome, and metabolite analyses of leaves, flowers, and developing fruits of Rutgers tomato infected with TPMVd.

In summary, our results contribute to the expanding knowledge that viroid infection in tomato leads to specific changes in reproductive gene expression. The interaction of silencing and signaling pathways and the activation of plant immune response affects plant growth and fruit development by a combination of arrest of ovary growth, aborted fruit, and male sterility in viroid-infected plants. Future studies to dissect the reproductive structures in tomato flowers in the viroid-infected tomato for examination of gene expression may reveal the extent to which the pattern of gene expression and the interaction of these pathways result in the activation or silencing of distinct genes in ovules and anthers.

## Supplementary Materials

The following are available online at http://www.mdpi.com/1999-4915/10/10/516/s1, Table S1: Oligonucleotide primer sets used in RT-PCR, real-time and stem-loop RT-PCR analyses. Table S2. List of network components shown in Figure S2. Figure S1: Rod-like secondary structures of TPMVd and the OG1 strain of MPVd. Figure S2: Description of components of the predicted network association of genes.
