# Supplementary material for: Pospiviroid Infection of Tomato Regulates the Expression of Genes Involved in Flower and Fruit Development
Source: Viruses. 2018 Sep 21;10(10):516. doi: 10.3390/v10100516 (PMC6213050; doi:10.3390/v10100516)
Supplement: Supplementary file 1 [file viruses-10-00516-s001.zip › Supplementary Files/Supplementary Material Table S2 Avina-Padilla et al..pdf]

Aviña-Padilla *et al.*

Supplementary Table S2. List of network components shown in Supplementary Figure S2.

Input Genes:

Abbreviation	Gene ID	GO:Description/Function	Protein Size
APETALA 1/MADS-MC	Solyc05g056620	MADS-box transcription factor MADS-MC	(244 aa)
ARF6b	Solyc00g196060	Auxin response factor 6	(354 aa)
ARF8a	Solyc02g037530	Auxin response factor 8; Auxin response factors (ARFs) are transcriptional factors that binds specifically to the DNA sequence 5'-TGTCTC-3' found in the auxin-responsive promoter elements (AuxREs)	(842 aa)
DWF-1	Solyc02g030170	DWARF1/DIMINUTO Plays a critical role in the general process of plant cell elongation. Involved in the synthesis of campesterol, an early precursor of brassinolide. Regulate indirectly phytochrome-mediated light responses through the modulation of brassinosteroid biosynthesis	(568 aa)
Fad7	Solyc06g051400	Omega-3 fatty acid desaturase	(435 aa)
Fw2.2	Solyc02g090730	ORFX Fw2.2; ORFX Fruit Weight	(163 aa)
GAI	Solyc11g011260	DELLA protein GAI; Probable transcriptional regulator that acts as a repressor of the gibberellin (GA) signaling pathway. Probably acts by participating in large multiprotein complexes that repress transcription of GA- inducible genes. Upon GA application, it is degraded by the proteasome, allowing the GA signaling pathway. Its degradation is not essential for germination	(588 aa)
GA20ox1/3OH-1	Solyc06g066820	3b-hydroxylase. Converts the inactive gibberellin precursors GA9 and GA20 in the bioactives gibberellins GA4 and GA1.	(374 aa)
GA7ox	Solyc06g069900	1-aminocyclopropane-1-carboxylate oxidase.– gibberellin 20-oxidase activity, gibberellin 2-beta-dioxygenase activity	(314 aa)
GIB1	Solyc08g006770	Anthocyanidin synthase oxoglutarate and iron dependent oxygenase	(351 aa)
PKv	Solyc12g017390	Viroid symptom modulation protein. Serine/threonine protein kinase	(467 aa)
SIAG6	Solyc01g093960	MADS box transcription factor	(252 aa)
SIIAA3	Solyc09g065850	IAA3; Aux/IAA proteins are short-lived transcriptional factors that function as repressors of early auxin response genes at low auxin concentrations	(185 aa)
SIIAA9/ctd1	Solyc04g076850	IAA16; Aux/IAA proteins are short-lived transcriptional factors that function as repressors of early auxin response genes at low auxin concentrations	(251 aa)
SIGRAS24	Solyc01g090950	GRAS family transcription factor (AHRD V1 **-- B9GPD0_POPTR); contains Interpro domain(s) IPR005202 GRAS transcription factor	(102 aa)
SIWUS	Solyc02g083950	Protein WUSCHEL; Transcription factor that plays a central role during developmental processes such as early embryogenesis and flowering, probably by regulating expression of specific genes. Required to specify stem cell identity in meristems, such as shoot apical meristem (SAM). May induce shoot stem cells activity in order to maintain the stem cell identity (By similarity)	(272 aa)
SlOVA6	Solyc01g096870	Prolyl-tRNA synthetase. Protein Binding. Glutamate t-RNA ligase activity.	(547 aa)
SlBigPetal1	Solyc05g006650	SlbHLH036. BHLH Transcription factor, similar to BigPetal1 in Arabidopsis. Sequence-specific DNA binding transcription factor activity	(272 aa)
TAG1	Solyc02g071730	Floral homeotic protein AGAMOUS; Probable transcription factor involved in regulating genes that determines stamen and carpel development in wild-type flowers	(248 aa)
YABBY1	Solyc11g071810	Fasciated, FAS protein. Transcription factor, The fasciated (fas) gene encodes a YABBY-like transcription factor whose downregulation during flower development causes high locule number, extreme large fruit size, in tomato fruit. The mutation is caused due to a 6 - 8 kb insertion in the first intron of the locus.	(177 aa)

Functional Partners:

20ox-1	Solyc03g006880	Gibberellin 20-oxidase-1, Catalyzes the oxidation of GA53 and GA19 to GA44 GA20 and GA19, precursors of the GA1 (active form). AF049898	(378 aa)
20ox-3	Solyc11g072310	AF049900, Gibberellin 20-oxidase-3. Catalyzes the oxidation of GA53 and GA19 to GA44 GA20 and GA19, precursors of the GA1 (active form).	(379 aa)
3BH-isomerase	Solyc06g082980	3-beta-hydroxysteroid-Delta8 Delta7-isomerase (AHRD V1 **-* C3KH60_ANOFI); contains Interpro domain(s) IPR007905 Emopamil-binding. C-8 sterol isomerase activity	(221aa)
ADP R Factor	Solyc07g006640	ADP-ribosylation factor 3 (AHRD V1 ***- B4FM64_MAIZE); contains Interpro domain(s) IPR006688 ADP-ribosylation factor. GTP binding, intracellular	(182aa)
asr	Solyc02g080640	Adenylyl-sulfate reductase asr	(461 aa)
CLV2	Solyc04g056640	Leucine rich repeat receptor like protein CLAVATA2	(746 aa)

Coi1	Solyc05g052620	Coronatine-insensitive 1	(603 aa)
CRY1	Solyc04g074180	Cryptochrome 1	(679 aa)
CRY2	Solyc09g090100	Cryptochrome 2	(635 aa)
CYP51	Solyc01g008110	Obtusifoliol 14alpha-demethylase	CYP51
Cyt C	Solyc01g103220	Cytochrome c; Electron carrier protein. The oxidized form of the cytochrome c heme group can accept anelectron from the heme group of the cytochrome c1 subunit of cytochrome reductase. Cytochrome c then transfersthiselectrontothecytochromeoxidasecomplex,the nalproteincarrierinthemitochondrialelectron-transport chain	(112aa)
DMI1	Solyc10g044890	DMI1 protein (Fragment) (AHRD V1 *-*- A9DNI2_MEDTR); contains Interpro domain(s) IPR016040 NAD(P)-binding domain. Metabolic process	(799aa)
FALSIFLORA	Solyc03g118160	FLORICAULA/LEAFY-like protein	(429 aa)
FK	Solyc09g009040	Delta14-sterol reductase	(369 aa)
FRK1	Solyc03g006860	Fructokinase	(347 aa)
FRK2	Solyc06g073190	Fructokinase-2; May play an important role in maintaining the ux of carbon towards starch formation	(328 aa)
FRK3	Solyc02g091490	Fructokinase 3. Fructokinase activity	(389 aa)
G2BDO	Solyc04g008670	Oxoglutarate and iron-dependent oxygenase	(357 aa)
G2BDO7	Solyc02g080120	Gibberellin 2-beta-dioxygenase 7 Oxoglutarate and iron-dependent oxygenase	(380 aa)
GA2ox1	Solyc05g053340	Gibberellin 2-oxidase	(349 aa)
GA2ox2	Solyc07g056670	Gibberellin 2-oxidase 2	(322 aa)
GA2ox3	Solyc01g079200	Gibberellin 2-oxidase	(344 aa)
GA2ox4	Solyc01g093980	Gibberellin 20-oxidase 4	(364 aa)
GA2ox5	Solyc07g061720	Gibberellin 2-oxidase	(331 aa)
GDH	Solyc03g094010	Glutamate dehydrogenase (AHRD V1 **** Q94IA5_BRANA); contains Interpro domain(s) IPR014362 Glutamate dehydrogenase glutamate dehydrogenase [NAD(P)+] activity	(411 aa)
GDH1	Solyc06g033860	Glutamate dehydrogenase (AHRD V1 **** Q94IA5_BRANA); contains Interpro domain(s) IPR014362 Glutamate dehydrogenase glutamate dehydrogenase [NAD(P)+] activity	(411 aa)
GDH2	Solyc01g068210	Glutamate dehydrogenase (AHRD V1 ***- A2TQI4_9FLAO); contains Interpro domain(s) IPR006095 Glutamate/phenylalanine/leucine/valine dehydrogenase	(634 aa)
GID1	Solyc09g074270	Putative GID1-like gibberellin receptor	(345 aa)
Glucosidase	Solyc01g059980	Glucan endo-1,3-beta-glucosidase B ; Implicated in the defense of plants against pathogens	(360 aa)
Glutamate synthase (Ferredoxin)	Solyc03g063560	Glutamate synthase (Ferredoxin) (AHRD V1 ***- B8HTZ7_CYAP4); contains Interpro domain(s) IPR002932 Glutamate synthase, central-C	(1621aa)
GP91	Solyc08g081690	NADPH oxidase	(963 aa)
GPA1	Solyc08g061220	Guanine nucleotide-binding protein alpha-1 subunit ; Guanine nucleotide-binding proteins (G proteins) are involved as modulators or transducers in various transmembrane signaling systems	(384 aa)
HY5	Solyc08g061130	Transcription factor that promotes photomorphogenesis in the light and positively HY5 regulates fruit pigmentation and fruit nutritional quality. Probably acts downstream of the light receptor network and directly affects transcription of light- induced genes	(158 aa)
IAA9	Solyc01g097290	IAA16 ; Aux/IAA proteins are short-lived transcriptional factors that function as repressors of early auxin response genes at low auxin concentrations	(251 aa)
J	Solyc11g010570	MADS-box protein JOINTLESS; Putative transcription factor that coordinates gene expression underlying the J differentiation of the pedicel abscission zone. May also be involved in the maintenance of the in florescence meristem state	(265 aa)
kdsA	Solyc11g072690	3-desoxy-D-manno octulosonic acid-8-phosphate synthase	(290 aa)
Lanceolate	Solyc07g062680	Lanceolate	(400 aa)
LeANT	Solyc04g077490	AP2-like ethylene-responsive transcription factor ANT-like	(652 aa)
Lin5	Solyc09g010080	Beta-fructofuranosidase; Cell-wall invertase	(584 aa)
MA-1	Solyc01g099760	The 26S protease is involved in the ATP-dependent degradation MA-1 of ubiquitinated proteins. The regulatory (or ATPase) complex confers ATP dependency and substrate specificity to the 26S complex (By similarity) 26S protease regulatory subunit 6A homolog.	(423 aa)
MAPK10	Solyc10g007500	Mitogen-activated protein kinase 10	(593 aa)
MAPK12	Solyc07g056350	Mitogen-activated protein kinase 12	(621 aa)

MAPK16	Solyc12g040680	Mitogen-activated protein kinase 16	(575 aa)
NIA	Solyc11g013810	Nitrate reductase [NADH]; Nitrate reductase is a key enzyme involved in the rst step of nitrate assimilation in plants, fungi and bacteria	(912 aa)
nii2	Solyc10g050890	Nitrite reductase	(584 aa)
Nitrate transporter	Solyc03g094010	Major facilitator superfamily, general substrate transporter	(460 aa)
opr3	Solyc07g007870	12-oxophytodienoate reductase 3. Specifically cleaves oleonic bonds in cyclic enones. Involved in the biosynthesis of jasmonic acid (JA) and perhaps in biosynthesis or metabolism of other oxylipin signaling opr3 moleclules. It is required for the spatial and temporal regulation of JA levels during dehiscence of anthers, promoting the stomium degeneration program (By similarity). In vitro, reduces 9S,13S-12- oxophytodienoic acid (9S,13S-OPDA) and 9R,13R-OPDA to 9S,13S-OPC- 8-0 and 9R,13R-OPC-8-0, respectively	(396 aa)
Oratate phosphoribosyltransferase	Solyc11g068830	Orotidine 5&apos-phosphate decarboxylase (Orotate phosphoribosyltransferase) (AHRD V1 **** C4QAV6_SCHMA); contains Interpro domain(s) IPR001754 Orotidine 5'-phosphate decarboxylase, core	(477 aa)
phyA	Solyc10g044670	Phytochrome; Regulatory photoreceptor which exists in two forms that are reversibly interconvertible by light- phyA the Pr form that absorbs maximally in the red region of the spectrum and the Pfr form that absorbs maximally in the far-red region	(1123 aa)
pnk-1	Solyc05g005250	Partially similar to pantothenate kinase family protein. pantothenate kinase family protein AB211526	(426 aa)
Protein kinase A-like	Solyc05g005250	protein phosphorylation. protein serine/threonine phosphatase complex. catalytic activity cAMP-dependent protein kinase complex	(1080 aa)
PYD1	Solyc02g044040	Dihydroorotate dehydrogenase family protein, Aldolase-type TIM barrel	(250 aa)
PYD1A	Solyc03g097910	Putative dehydrogenase, dihydropyrimidine dehydrogenase (NADP+) activity	(429 aa)
PYRD	Solyc01g008290	Dihydroorotatedehydrogenase(quinone),mitochondrial	(457 aa)
RBCOA	Solyc09g011080	Ribulose-1 5-bisphosphate carboxylase/oxygenase activase 1 (AHRD V1 **** Q9AXG1_GOSHI); contains Interpro domain(s) IPR003959 ATPase, AAA-type, core	(439 aa)
RLPK	Solyc09g011060	Clade IV lectin receptor kinase. Receptor like protein kinase (AHRD V1 **** Q39139_ARATH); contains Interpro domain(s) IPR001220 Legume lectin, beta chain. protein binding kinase activity	(832 aa)
RNA P	Solyc12g035870	DNA-directed RNA polymerase subunit beta (AHRD V1 ***- D3WBK7_9AQUA); contains Interpro domain(s) IPR015712 DNA-directed RNA polymerase, subunit 2	(398 aa)
SlbHLH006	Solyc01g102300	SlbHLH006 is part of the 159 member basic helix-loop-helix gene family that is known to play important role in physiological, developmental and metabolic processes.	(715 aa)
SlbHLH052	Solyc07g043580	SlbHLH052 is part of the 159 member basic helix-loop-helix gene family that is known to play important role in physiological, developmental and metabolic processes.	(495 aa)
SlTPL1	Solyc03g117360	SlTPL1 belongs to the 6 member TOPLESS gene family. These genes are involved in gene repression in several mechanisms.	(2226 aa)
SlTPL2	Solyc08g076030	SlTPL2 belongs to the 6 member TOPLESS gene family. These genes are involved in gene repression in several mechanisms. WD-40 repeat protein (AHRD V1 *--- C7RS72_ACCPU); contains Interpro domain(s) IPR017986 WD40 repeat, region. protein homodimerization activity. transcription repressor activity	(1135 aa)
SPY	Solyc09g010180	Probable UDP-N-acetylglucosamine--peptide N-acetylglucosaminyltransferase SPINDLY; Probable O-linked N-acetylglucosamine transferase (OGT) involved in various processes such as gibberellin (GA) signaling pathway. spy OGTs catalyze the addition of nucleotide-activated sugars directly onto the polypeptide through O-glycosidic linkage with the hydroxyl of serine or threonine. Probably acts by adding O- linked sugars to yet unknown proteins	(931 aa)
T12	Solyc07g007120	Homeobox protein knotted-1-like LET12 ; May have a role to play in formative events in ovule and embryo morphogenesis	(431 aa)
T6	Solyc02g081120	Homeobox protein knotted-1-like LET6 ; May have a role to play in formative events in ovule and embryo morphogenesis. Probably binds to the DNA sequence 5'-TGAC- 3'	(354 aa)
TM29	Solyc02g089200	MADS-box protein. Transcription factor. TM29 belongs to the SEPALLATA (SEP) subfamily of the MADS-box gene family. Protein binding, sequence-specific DNA binding transcription factor activity	(246 aa)
TOMPRORNA prosystemin	Solyc05g051750	Activates a lipid-based signal transduction pathway in which linolenic acid is converted to jasmonic acid,a potent activator of defense gene transcription, including proteinase inhibitor	(201 aa)
TOMTRALTBE	Solyc01g009310	Meloidogyne-induced giant cell protein DB217, 7-dehydrocholesterol reductase activity, delta-7 reductase activity	(434 aa)
Unk	Solyc10g005470	Unknown, Uncharacterized protein	(139 aa)
VGT1	Solyc03g078000	Vacuolar glucose transporter 1	(545 aa)
VIN3	Solyc07g018270	Vernalization insensitive 3, Fibronectin, type III	(739 aa)
VIRP1	Solyc01g106280	PSTVd RNA-biding protein, Virp1; PSTVd RNA-binding protein Virp1a; PSTVd RNA-binding protein Virp1b; PSTVd RNA-binding protein Virp1c; PSTVd RNA-binding protein Virp1d	(602 aa)
ZWICHEL	Solyc02g068340	Kinesin-like calmodulin binding protein kinesin motor region, calmodulin binding	(1265 aa)

APETALA 1/MADS-MC	Solyc05g056620	MADS-box transcription factor MADS-MC	(244 aa)
ARF6b	Solyc00g196060	Auxin response factor 6	(354 aa)
ARF8a	Solyc02g037530	Auxin response factor 8; Auxin response factors (ARFs) are transcriptional factors that binds specifically to the DNA sequence 5'-TGTCTC-3' found in the auxin-responsive promoter elements (AuxREs)	(842 aa)
DWF-1	Solyc02g030170	DWARF1/DIMINUTO Plays a critical role in the general process of plant cell elongation. Involved in the synthesis of campesterol, an early precursor of brassinolide. Regulate indirectly phytochrome-mediated light responses through the modulation of brassinosteroid biosynthesis	(568 aa)
Fad7	Solyc06g051400	Omega-3 fatty acid desaturase	(435 aa)
Fw2.2	Solyc02g090730	ORFX Fw2.2; ORFX Fruit Weight	(163 aa)
GAI	Solyc11g011260	DELLA protein GAI; Probable transcriptional regulator that acts as a repressor of the gibberellin (GA) signaling pathway. Probably acts by participating in large multiprotein complexes that repress transcription of GA- inducible genes. Upon GA application, it is degraded by the proteasome, allowing the GA signaling pathway. Its degradation is not essential for germination	(588 aa)
GA20ox1/3OH-1	Solyc06g066820	3b-hydroxylase. Converts the inactive gibberellin precursors GA9 and GA20 in the bioactives gibberellins GA4 and GA1.	(374 aa)
GA7ox	Solyc06g069900	1-aminocyclopropane-1-carboxylate oxidase.– gibberellin 20-oxidase activity, gibberellin 2-beta-dioxygenase activity	(314 aa)
GIB1	Solyc08g006770	Anthocyanidin synthase oxoglutarate and iron dependent oxygenase	(351 aa)
PKv	Solyc12g017390	Viroid symptom modulation protein. Serine/threonine protein kinase	(467 aa)
SIAG6	Solyc01g093960	MADS box transcription factor	(252 aa)
SIIAA3	Solyc09g065850	IAA3; Aux/IAA proteins are short-lived transcriptional factors that function as repressors of early auxin response genes at low auxin concentrations	(185 aa)
SIIAA9/ctd1	Solyc04g076850	IAA16; Aux/IAA proteins are short-lived transcriptional factors that function as repressors of early auxin response genes at low auxin concentrations	(251 aa)
SIGRAS24	Solyc01g090950	GRAS family transcription factor (AHRD V1 **-- B9GPD0_POPTR); contains Interpro domain(s) IPR005202 GRAS transcription factor	(102 aa)
SIWUS	Solyc02g083950	Protein WUSCHEL; Transcription factor that plays a central role during developmental processes such as early embryogenesis and flowering, probably by regulating expression of specific genes. Required to specify stem cell identity in meristems, such as shoot apical meristem (SAM). May induce shoot stem cells activity in order to maintain the stem cell identity (By similarity)	(272 aa)
SlOVA6	Solyc01g096870	Prolyl-tRNA synthetase. Protein Binding. Glutamate t-RNA ligase activity.	(547 aa)
SlBigPetal1	Solyc05g006650	SlbHLH036. BHLH Transcription factor, similar to BigPetal1 in Arabidopsis. Sequence-specific DNA binding transcription factor activity	(272 aa)
TAG1	Solyc02g071730	Floral homeotic protein AGAMOUS; Probable transcription factor involved in regulating genes that determines stamen and carpel development in wild-type flowers	(248 aa)
YABBY1	Solyc11g071810	Fasciated, FAS protein. Transcription factor, The fasciated (fas) gene encodes a YABBY-like transcription factor whose downregulation during flower development causes high locule number, extreme large fruit size, in tomato fruit. The mutation is caused due to a 6 - 8 kb insertion in the first intron of the locus.	(177 aa)
