# Supplementary material for: Pospiviroid Infection of Tomato Regulates the Expression of Genes Involved in Flower and Fruit Development
Source: Viruses. 2018 Sep 21;10(10):516. doi: 10.3390/v10100516 (PMC6213050; doi:10.3390/v10100516)
Supplement: Supplementary file 1 [file viruses-10-00516-s001.zip › Supplementary Files/Supplementary Material-Figure S1 Avina-Padilla et al..pdf]

Aviña-Padilla et al.

Supplementary Figure 1.

Rod-like secondary structures of tomato planta macho viroid (TPMVd; GenBank K00817) and the OG1 strain of Mexican papita viroid (MPVd; GenBank L78454) predicted using the UNAFold Web Server (http://unafold.rna.albany.edu/?q=mfold ; Zuker, M. *Nucleic Acids Res.* **2003**; 31 (13), 3406-3415.). The thermodynamically most stable structures are shown. The putative functional domains are indicated (Keese, P.; Symons, R. H. **1985**. *Proc. Natl. Acad. Sci. USA*. 82, 4582-4586). T_L= terminal left; T_R = terminal right. The nucleotide differences between MPV-OG1 and TPMVd are highlighted in red. T_L= terminal left; T_R = terminal right. The T_R domain containing a single base pair ($_{174}$G:C$_{183}$; shown in blue) determining virulence in TPMVd is boxed (Li, R.; Padmanabhan, C.; Ling, K-S. **2017**. *Virology* 500, 238-246.)

TPMVd ΔG=-152.20

```
 1                                         40                                          80                                         120                                       160
 CG          CCUUGU      UGU   ACA-     GAC    U   -   AA    AAAA     UU-     CAAA-   GC       GGA--   -     GAAAC-        A  U    A     UC  -   U    A   U-  -     CA    UAAUC    CU    C--     U
    GGAUCUUUU      GGUUCC   GGU    CACCU   CUCC GA CCAG  AAGA    AGAA   GCGGC      GGA  GCUUCAG     UCC CCGGG      CUGGAGCG AC GGCG AGGAG  GC GGC GGGG GUC  CC CAGA   GGAG     CCCG  GAAA   AGGGU U
    CCUGGGAAA      CCAAGG   CCA    GUGGG   GGGG CU GGUC  UUCU    UCUU   CGCCG      CCU  CGAAGUC     AGG GGCCC      GGCUUCGC UG UCGC UUCUC  CG CCG UCCC CAG  GG GUCU   CCUU     GGGC  CUUU   UCCCA U
 UC          AGCUUU      UC-   AAAG     AGC    C   C   G-    AUC-     UUU     CGAAC   --       AACAA   U     AUCAGA        -  -    G     UU  C   C    A   CU  C     --    CCUUU    UU    CCU     C
360                                     320                                    280                                              240                                                            180
```

T_L Pathogenicity Central Conserved Variable T_R

MPVd-OG1 ΔG=-160.20

```
 1                                          40                                            80                                       120                                       160
 CG       UU   UU   -     UGU    CA-     GAC     A  -    GA    AAAAA         --     A  AGC   -   GGA--         GAAAC-         A  U  AAA     UC    -  U    A   U-  U    CA    UAAUC    CU    C--     U
    GGAUCU  UCC  GU GGUUCC   GGUU   CACCU    CUCC GC CCAG  AAGA     GAAAGGCGG  CUCGG GG   GCUUCAG     UCC CCGGG      CUGGAGCGA C GGC    GGAG  GCGG C  GGGG GUC  CC CAGA GGAG     CCCG  GAAA   AGGGU U
    CCUGGG  AGG  CG CCAAGG   CCGA   GUGGG    GGGG CG GGUC  UUCU     UUUUUCGCC  GAGCC CU   CGAAGUC     AGG GGCCC      GGCUUCGCU G UCG    UCUC  CGCC G  UCCC CAG  GG GUCU CCUU     GGGC  CUUU   UCCCA U
 UC       U-   -U   C     UC-    AAG     AGC     A  C    G-    AUCUC         AA     -  ---       AACAA   U     AUCAUA         -  -  CG-     UC    C  C    A   CU  U    --    CCUUU    UU    CCU     C
360                                      320                                   280                                              240                                                               180
```

T_L Pathogenicity Central Conserved Variable T_R
