# Supplementary material for: Pospiviroid Infection of Tomato Regulates the Expression of Genes Involved in Flower and Fruit Development
Source: Viruses. 2018 Sep 21;10(10):516. doi: 10.3390/v10100516 (PMC6213050; doi:10.3390/v10100516)
Supplement: Supplementary file 1 [file viruses-10-00516-s001.zip › Supplementary Files/Supplementary Material-Figure S2 Avina-Padilla et al..pdf]

Aviña-Padilla et al.

Supplementary Figure S2. Description of components of the predicted network association of genes. Network nodes represent proteins, splice isoforms or post-transcriptional modifications; each node represents all the proteins produced by a single protein coding gene locus. Edges represent protein-protein associations; associations are specific and meaningful; proteins jointly contribute to a similar function, not necessarily meaning, they have physical bindings. Predictions were made using STRING https://string-db.org with gene ontology (GO) enrichment.

Node Color

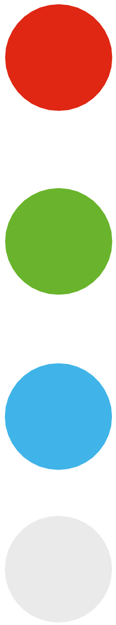

-Over-expressed genes. Query proteins and first shell of interactors.

-Repressed genes. Query proteins and first shell of interactors.

-No changes in expression.

-Second shell of interactors.

Edge Color

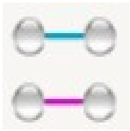

-From Curated Databases

-Experimentally determined

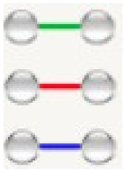

-Gene Neighborhood

-Gene Fusions

-Gene Co-occurrence

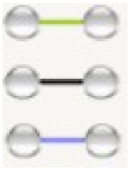

-Text-mining

-Co-expression

-Protein Homology

Current Organism

Solanum lycopersicum
NCBI taxonomy Id: 4081

Other names: *Lycopersicon, Lycopersicon (Mill.) Seithe, Lycopersicon esculentum, Lycopersicon esculentum Mill., Lycopersicon esculentum var. esculentum, Lycopersicon lycopersicum, S. lycopersicum, Solanum esculentum, Solanum esculentum Dunal, Solanum lycopersicum.*

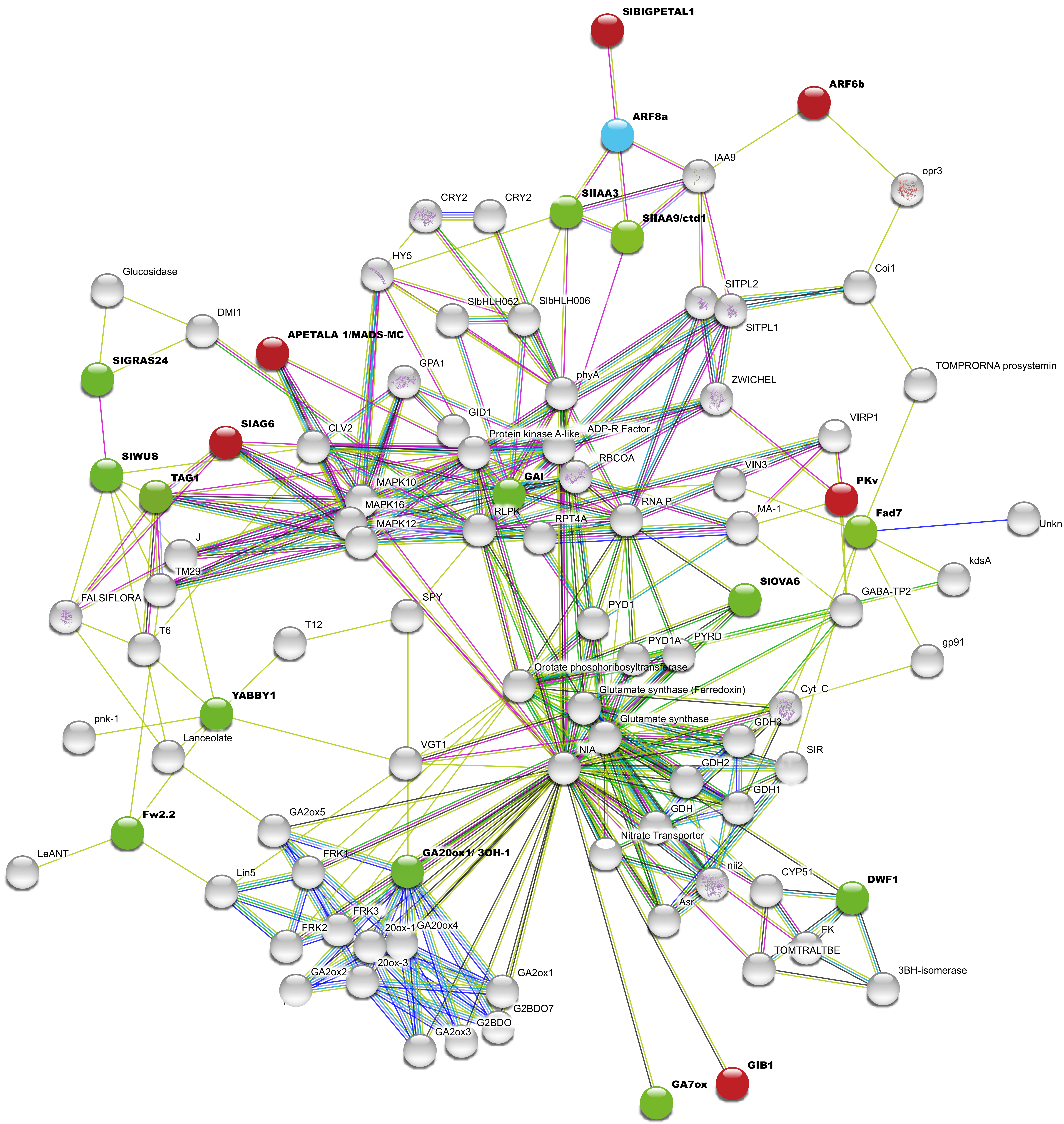

SlBIGPETAL1
ARF6b
ARF8a
IAA9
opr3
CRY2
CRY2
SlIAA3
SlIAA9/ctd1
HY5
Glucosidase
Coi1
SlTPL2
SlbHLH052
SlbHLH006
DMI1
SlTPL1
APETALA 1/MADS-MC
SlGRAS24
GPA1
phyA
TOMPRORNA prosystemin
ZWICHEL
GID1
VIRP1
SlAG6
CLV2
Protein kinase A-like
ADP-R Factor
SlWUS
RBCOA
TAG1
MAPK10
GAI
VIN3
PKv
MAPK16
RNA P
RLPK
MA-1
Fad7
MAPK12
RPT4A
Unkn
J
kdsA
TM29
SlOVA6
FALSIFLORA
SPY
GABA-TP2
PYD1
T12
T6
PYD1A
PYRD
gp91
Orotate phosphoribosyltransferase
Glutamate synthase (Ferredoxin)
YABBY1
Cyt C
pnk-1
Glutamate synthase
GDH3
Lanceolate
VGT1
NIA
SIR
GDH2
GDH1
GA2ox5
GDH
Fw2.2
Nitrate Transporter
LeANT
FRK1
GA20ox1/ 3OH-1
Lin5
nii2
CYP51
DWF1
FRK3
Asr
FRK2
20ox-1
GA20ox4
FK
TOMTRALTBE
20ox-3
GA2ox2
GA2ox1
3BH-isomerase
G2BDO7
G2BDO
GA2ox3
GIB1
GA7ox
