# Supplementary material for: Pospiviroid Infection of Tomato Regulates the Expression of Genes Involved in Flower and Fruit Development
Source: Viruses. 2018 Sep 21;10(10):516. doi: 10.3390/v10100516 (PMC6213050; doi:10.3390/v10100516)
Supplement: Supplementary file 1 [file viruses-10-00516-s001.zip › Supplementary Files/Supplementary Material-Table S1 Avina-Padilla et al..pdf]

Table 1S. Oligonucleotide primer sets used in RT-PCR, real-time and stem-loop RT-PCR analyses.

Gene	Oligonucleotide	Sequence (5’–3’)	Gene, Accession No, or Gene Locus Identity	Reference[a]
Pospiviroid	POSPI-FW/POSPI-RE	GGGATCCCCGGGGAAAC/ AGCTTCAGTTGTWTCCACCGGGT		Verhoeven *et al.*, 2004
bHLH TF (*BIGPETAL1* homolog)	BHLH tomato_F/ BHLH tomato_R	GCAGCAAACATTCGAGACAA/ GCCTTTGATGCCTCCAATTA	*SlBIGPETAL1*; Tomato_SGNlocusID_20267_Solyc04g005130	this paper
Proline-like t-RNA synthetase (OVA6) (*OVULEABORTION6* homolog)	Ovule Abortion tomato_F/ Ovule Abortion tomato_R	ATTGTCGGCGTTTTAGTTGG/ GCTGCTTGATGGCAGTTACA	*SlOVA6;* Tomato_SGNlocusID_12237_Solyc01g096870	this paper
EF-1α	EF1-F / EF1 R	GATTGGTGGTATTGGAACTGTC/ GCTTCGTGGTGCATCTC	*S. lycopersicum* elongation factor 1 (*EF-1*); X14449	Hammond and Zhao, 2009
expansin	EXPF/EXPR	TGTTGGAGGTGCTGGTGATA/ CCCCTCAAAAGTTTGTCCA	*LeEXP2*; AF096776	Hammond and Zhao, 2009
pkv (protein kinase viroid induced)	PKVRQF/PKVRQR	TCCGTTGTTCTGTCAATCCA/ CCTCCCACAAAAAGACCAAA	*pkv;* EU196240	Hammond and Zhao, 2009
GA20ox1	GA20oxRQF/GA20ox RQR	TTCCGGTTCCACTTATCGAC/ GGCGTTGGAGATGATATTA	*GA20oxidase1*; AB032198	Hammond and Zhao, 2009
GA7ox	GA7OXF/GA7OXR	TTTGAGCACTTGTGGTTTCG/TGCTTG AAATAAGGTGTAATGCTG	*GA7 oxidase*	Katsarou *et al.*, 2016
SlIAA3	SlIAA3F/SlIAA3R	GTTAGCATGGATGGAGCACC/ACCAA CAAGCATCCAATCAAC	Tomato AUX/IAA transcription factor; JN379433	this paper
IAA9	R-IAASolF/ R-IAASolF	GCTGTGAATGATGCCTCAAA/ TCAAATAGGGAGCACCATCC	AUX/IAA9 transcriptional regulator; AJ937282	this paper
ARF6A	ARF6ASolF/ ARF6ASolR	TTTCATGAACCGGAACCATT/ CAAAATTGCCAACGCGTGTG	Auxin responsive factor 6A *Solyc00g196060*	Liu *et al.*, 2016
ARF8B	ARF8BSolF/ ARF8BSolR	GGGAAAGGAAGAGGCTGAAT/ CGAAAGCTAAAGAAGCCAGGT	Auxin responsive factor 8B *Solyc02g037530*	Liu *et al.*, 2016

SlAG6	SlAGL6F/SlAGL6R	AAACCCTTGAGAGGTACCAACG/ CACCAAGCAAGTGCCTTTGAG	*Solanum AGAMOUS*-like 6 MADS-box-transcription factor *Solyc01g093960*	Klap *et al.*, 2017
SlGRAS24	qSlGRAS24F/ qSlGRASR	TGGTCCAGACAGGGAATCCG/ TTCAAGTTGTTGGTGTTGGCA	Transcription factor; *Solyc01g090950.2*; SGN_U569651	Huang *et al.*, 2017
SlDELLA	qSlDELLAF/ qSlDELLAR	TGATGCGACTATACTTGATATAAG/ GGGTTAATCTGTTTAATAGAGTTC	*Solyc11g011260.1*; SGN_U575114	Huang *et al.*, 2017
Dwarf1-DIMINUTO	Dwarf1-DIMF/ Dwarf1-DIMR	TCCAAGATGTCGGATGCTAA/ GATCCATCGGAACGAAAGAA	Protein involved in steroid synthesis *Solyc02g030170*	Katsarou *et al.*, 2016
SlWUS	SlWUSF/SlWUSR	CCAGCAACTTACCCTTTTCTTG/TAAA GCAGAGTTACCCCTTTGG	*WUSCHEL; Solyc02g083950*	Li *et al*, 2017b
YABBY	YABBYF/YABBYR	GTAAGATGTGGGCATTGTGC/TGTACT GTTGCCTTTGCAGC	Zinc-finger domain protein*; Solyc11g071810*	Li *et al.*, 2017b
FW2.2	FW2.2F/FW2.2R	GCTTAAGAACCGTGGCTTTG/TGATAA GGGGGCATGGTAAC	*FRUIT WEIGHT 2.2, Solyc02g090730*	Li *et al.*, 2017b
APETALA1	ApetalaF/ApetalaR	TGCGATGCTGAAGTTGCTTT/GTTTTC CTGCACCGGTGATT	Sol Apetala-like MADS box transcription factor; AY306154	this paper
TAG1	TAG1F/TAG1R	CTTGATGCCAGGGAGTTCAT/ ATCGAATTGCTGAGGTGGAG	Tomato agamous-like transcription factor; Solyc*02g071730*	Li et al., 2017b
miR156	RT primer/Forward primer	GTCGTATCCAGTGCAGGGTCCGAGGT ATTCGCACTGGATACGACGTGCTC/GC GGCGGTGACAGAAGAGAGT		Varkonyi-Gasic *et al.*, 2010
miR159	RT primer/Forward primer	GTCGTATCCAGTGCAGGGTCCGAGGT ATTCGCACTGGATACGACTAGAGC/C GGCGGTTTGGATTGAAGGGA		Varkonyi-Gasic *et al.*, 2010
miR167	RT primer/Forward primer	GTCGTATCCAGTGCAGGGTCCGAGGT ATTCGCACTGGATACGACCTAGATC/T CGCGTGAAGCTGCCAGCAT		Varkonyi-Gasic *et al.*, 2010
miR171	RT primer/Forward primer	GTCGTATCCAGTGCAGGGTCCGAGGT ATTCGCACTGGATACGACGATATT/TT CCTTGATTGAGCCGCGCC		Varkonyi-Gasic *et al.*, 2010
miR396	RT primer/Forward primer	GTCGTATCCAGTGCAGGGTCCGAGGT ATTCGCACTGGATACGACCAGTTC/CG GCGTTCCACAGCTTTCTT		Varkonyi-Gasic *et al.*, 2010
miR408	RT primer/Forward primer	GTCGTATCCAGTGCAGGGTCCGAGGT ATTCGCACTGGATACGACGCCAGG/C GGCGCATGCACTGCCTCTTC		Varkonyi-Gasic *et al.*, 2010

Universal miRNA reverse primer	Universal miRNA reverse primer	GTGCAGGGTCCGAGGT		Varkonyi-Gasic *et al.*, 2010
U6snRNA	U6snRNAF/ U6snRNAR	TCTAACAGTGTAGTTTGTCCCTTCG/T TGTGCGTGTCATCCTTGC	U6 small nuclear RNA	Feng *et al.*, 2013.

[a] Feng, J., Lin, R., and Chen, J. (2013). *Mol. Biol. Rep.* 40, 3713-3722; Hammond, R.W., and Zhao, Y. (2009) *BMC Plant Biol* 9,108; Huang, W., Peng, S., Xian, Z., Lin, D., Hu, G., Yang, L., Ren, M., and Li, Z. (2017) *Plant Biotech. J.* 15, 472-488; Katsarou, K., Wu, Y., Zhang, R., Bonar, N., Morris, J., Hedley, P. E., Bryan, G. J., Kalantidis, K., and Hornyik, C. (2016) *PLoS One* 3:11(3): e0150711; Klap, C., Yeshayahou, E., Bolger, A. M., Arazi, T., Gupta, S. K., Shabtai, S., Usabel, B., Salts, Y., and Barg, R. (2017) *Plant Biotech. J.* 15, 634-647; Li, H., Qi, M., Sun, M., Liu, Y., Liu, Y., Xu, T., Li, Y., and Li, T. (2017b) *Front. Plant Sci.* 8: 457; Liu, X., Xu, T., Dong, X., Liu, Y., Liu, Z., Shi, Z., Wang, Y., Qi, M., and Li, T. (2016) *Acta Physiol. Plant* 38:77; Varkonyi-Gasic, E., Wu, R., Wood, M., Walton, E. F., and Hellens, R. P. (2007) *Plant Meth*. 3, 12; Verhoeven, J., Jansen, C., Willemen, T.M., Kox, L.F.F., Owens, R.A., and Roenhorst, J.W. (2004) *Eur. J. Plant Pathol.* 110, 823-831.
